# Special Issue on “Insights on Ecotoxicological Effects of Anthropogenic Contaminants in Aquatic Organisms”

**DOI:** 10.3390/toxics11040311

**Published:** 2023-03-27

**Authors:** Rosa Bonaventura, Francesca Zito, Roberta Russo

**Affiliations:** Istituto per la Ricerca e l’Innovazione Biomedica, Consiglio Nazionale delle Ricerche, Via Ugo La Malfa 153, 90146 Palermo, Italy

In human history, many key points have characterized technological progress, such as the use of metals, which began in prehistoric times and continues to the present day, with many industrial uses [1], or the domination of plastics, which started at the beginning of the 20th century, with disparate applications in various sectors [2]. Several human activities cause the release of industrial, agricultural, hospital, and domestic wastes into the sea as well as coastal and transitional ecosystems (estuaries and coastal lagoons) with exponential environmental impacts [3]. The competent authorities of many countries have engaged in the effort of reducing the anthropogenic impacts on these aquatic ecosystems and, indeed, since the 1960s, an evident reduction in the sediment metal burden has been observed [4]. Nevertheless, we still have to face new emerging pollutants, such as microplastics, pharmaceuticals, and personal care products (PPCPs) (drugs, chemicals, etc.), xenobiotics, such as pesticides (insecticides, herbicides, and fungicides), and endocrine-disrupting compounds (EDCs) [5,6]. All these substances show great persistence in the environment, can be toxic with direct effects on aquatic organisms, and, by accumulating in their tissues, can also compromise human health via the food chain [7]. In this respect, ecotoxicological studies, from field campaigns to laboratory experiments, are increasingly needed to better understand and expand our knowledge on the impacts caused by all these different pollutants, also acting as mixtures, at different levels on aquatic biota. 

The research and review articles collected in this Special Issue give contribute to this, presenting studies using different types of aquatic organisms, ranging from bacteria to small fish, that can be used to evaluate the impact of many anthropogenic contaminants, suggesting their use as bioindicators of aquatic pollution in future potential applications. Here, we summarize the contents of this Special Issue starting from the least evolved organism up to the vertebrates.

Through a field campaign study and metagenomics, authors investigated how anthropogenic abiotic features may affect the microbial composition, i.e., Archaea and Bacteria, and the antibiotic resistance genes (ARGs) and heavy metal resistance gene (HMRG) profiles, starting from surface sea water sampled from 21 different sites along the Portuguese mainland and the islands’ coastal and transitional systems [8].

Two interesting species of primary producers, the microalgae *Phaeodactylum tricornutum* and the macroalgae *Ulva lactuca*, were used to evaluate the effects of one of the most used anionic surfactants, Sodium Dodecyl Sulfate (SDS), commonly used in cleansing formulations [9]. A wide range of biochemical and biophysical assays were applied to evaluate the effects of environmentally relevant SDS concentrations on the two selected species, encouraging their use as suitable models for ecotoxicological assessments. 

Other authors reviewed the mechanisms adopted by different species of microalgae to cope with high copper (Cu) concentrations, as well as the potential use of microalgae for Cu bioremediation in aquatic environments [10]. Cu is an essential heavy metal playing a key role in many biological processes as it is a co-factor for Cu-dependent enzymes, but it can be harmful for a wide range of living organisms at elevated levels.

A marine zooplanktonic copepod species, *Acartia tonsa*, was used to provide a first screening on the toxicity of five synthetic commercially available Neonicotinoid (NEO) pesticides, analyzing their larval development. To fight against pest insects, the neurotoxic pesticide NEOs are widely used in agriculture and are easily transported into aquatic systems, due to their high water solubility, low soil adsorption, and through runoff and drainage from farmland. NEOs can affects many organisms, including copepods, which are marine invertebrate Crustaceans, showing many nervous system similarities to insects [11].

Using both traditional and novel techniques, another two zooplankton species, the brine shrimp *Artemia franciscana* and jellyfish *Aurelia* sp., were used to evaluate the ecotoxicity of polyvinylidene difluoride (PVDF) and polylactic acid (PLA) microplastics, two polymers having a wide array of applications in many fields [12].

Through a high-throughput screening approach, the molecular response of *Paracentrotus lividus* sea urchin embryos exposed to metals, i.e., lithium, nickel, and zinc, was characterized. Different types of human activities can release these metals into the environment where they can affect aquatic organisms. In nature, sea urchin embryos are part of the zooplankton and, since the birth of experimental embryology, they have been one of the best studied model organisms in ecotoxicological studies [13].

Climbing the evolutionary ladder, a well-known and important model in experimental embryology is the zebrafish embryo, *Danio rerio*. Here, it was used to evaluate the exposure and co-exposure to pollutants as potassium perchlorate (KClO_4_) and cadmium (Cd). Morphological alterations, mRNA expression related to thyroid function and oxidative stress, thyroid hormone levels, and malondialdehyde were measured in the exposed zebrafish embryos [14].

A field study, conducted along Croatian coastal areas in the Adriatic Sea, reported chemical and ecotoxicological evaluation, i.e., phytotoxicity test of marine sediments collected in five sampling sites, giving a contribution to the development of Croatian national criteria for sediment evaluation [15].

Impacts to marine organisms around Fukushima Daiichi Nuclear Power Plant after the 2011 Great East Japan Earthquake, and subsequent tsunami and nuclear disaster, were discussed in a review, taking into consideration the existing knowledge about the effects of radionuclides and ionizing radiation on aquatic organisms, such as many invertebrates and fishes [16].
